# Quantitative Analysis of High-Price Rice Adulteration Based on Near-Infrared Spectroscopy Combined with Chemometrics

**DOI:** 10.3390/foods13203241

**Published:** 2024-10-11

**Authors:** Mengting Chen, Jiahui Song, Haiyan He, Yue Yu, Ruoni Wang, Yue Huang, Zhanming Li

**Affiliations:** 1 School of Grain Science and Technology, Jiangsu University of Science and Technology, Zhenjiang 212100, China; 2 College of Food Science and Nutritional Engineering, China Agricultural University, Beijing 100083, China

**Keywords:** near-infrared spectroscopy, adulteration, back-propagation neural network, competitive adaptive reweighted sampling, support vector regression

## Abstract

Near-infrared spectroscopy (NIRS) holds significant promise in detecting food adulteration due to its non-destructive, simple, and user-friendly properties. This study employed NIRS in conjunction with chemometrics to estimate the content of low-price rice flours (Nanjing, Songjing, Jiangxi silk, Yunhui) blended with high-price rice (Wuchang and Thai fragrant). Partial least squares regression (PLSR), support vector regression (SVR), and back-propagation neural network (BPNN) models were deployed to analyze the spectral data of adulterated samples and assess the degree of contamination. Various preprocessing techniques, parameter optimization strategies, and wavelength selection methods were employed to enhance model accuracy. With correlation coefficients exceeding 87%, the BPNN models exhibited high accuracy in estimating adulteration levels in high-price rice. The SPXY-SG-BPNN, SPXY-MMN-BPNN, KS-SNV-BPNN, and SPXY-SG-BPNN models showcased exceptional performance in discerning mixed Wuchang japonica, Thai fragrant indica, and Thai fragrant Yunhui rice. As shown above, NIRS demonstrated its potential as a rapid, non-destructive method for detecting low-price rice in premium rice blends. Future studies should be performed to concentrate on enhancing the models’ versatility and practical applicability.

## 1. Introduction

Recently, the growing demand for high-value agricultural products has intensified the need for efficient and rapid technologies to ensure the authenticity and quality of rice, especially in the face of widespread supply chain fraud [1,2]. This fraud not only undermines consumer trust but also poses significant risks to public health, as adulterated products may contain harmful contaminants or allergens. Furthermore, such practices can distort market competition, penalizing honest producers and eroding the overall integrity of the food industry. Therefore, there is a critical need for advanced and reliable methods to detect and prevent the adulteration of rice, ensuring that consumers receive safe, authentic, and high-quality products. Traditional approaches, such as morphological and color-based chemical analysis, molecular biology techniques, and electronic tongue assessments, have been employed for commercial rice variety detection [3,4,5]. However, these methods face limitations, including high time and labor costs, complexity, high costs for molecular biology, and interference, instability, and maintenance expenses for electronic tongues.

In contrast, near-infrared spectroscopy (NIRS) is distinguished by its non-destructive nature, simplicity, high reliability, multi-analytical capabilities, and short detection times [6,7]. It has already shown its utility in various fields such as drug detection, monitoring of biological processes, food safety analysis, and the detection of food components, highlighting its potential as an efficient alternative to traditional methods [8]. The combination of NIRS with chemometrics holds great promise for the detection of food adulteration and has broad applicability [9]. Partial least squares regression (PLSR) and support vector regression (SVR) are powerful tools for spectral data analysis, known for their ability to handle complex, high-dimensional data and nonlinear relationships [10]. The integration of NIRS with PLSR and SVR can fully leverage their respective strengths, creating a comprehensive analytical system [11,12].

In practical applications, the spectral data produced by NIRS contains a vast amount of wavelength information, but only a subset of these wavelengths have a significant relationship with the target analytes. Therefore, performing wavelength variable selection is essential to eliminate redundant wavelengths, thereby enhancing the model’s stability and predictive accuracy. To achieve effective wavelength variable selection, competitive adaptive reweighted sampling (CARS) and successive projections algorithm (SPA) are commonly used and proven methods [13,14]. CARS reduces irrelevant and redundant wavelengths through an iterative process, ultimately identifying the wavelengths that contribute most to the model. SPA uses geometric methods to sequentially project and select wavelengths, ensuring minimal redundancy among the chosen wavelengths. The use of CARS and SPA significantly reduces redundancy in NIRS data, thereby enhancing the model’s simplicity and predictive performance.

In addition, a back-propagation neural network (BPNN), a multi-layer feedforward neural network trained with back-propagation, was also employed in this study [15,16,17]. Previous studies have combined NIRS with olfactory visualization data to measure the fatty acid content in rice. Based on these combined features, a BPNN detection model was developed, achieving an R^2^p of 0.926 [18,19]. Additionally, NIRS in conjunction with machine learning was used to determine the TVB-N content in various types of frozen grass carp fillets, where the OSC+D1-CARS-PSO-BP model demonstrated strong predictive capability, with an R^2^p of 0.987 [20]. BPNN is capable of solving combinatorial optimization and decision-making problems, features strong self-learning abilities and adaptability, and possesses powerful nonlinear mapping capabilities to learn and model complex nonlinear relationships [20,21,22].

In this study, spectral data from Northeast Wuchang (*Oryza sativa* subsp. *japonica*) and Thai fragrant (*Oryza sativa* subsp. *indica*) rice samples were divided using sample set partitioning based on joint x-y distance (SPXY) and Kennard–Stone (KS) algorithms, followed by six preprocessing techniques, including maximum-minimum normalization (MMN), multiple scattering correction (MSC), standard normal variable (SNV), convolution smoothing, Savitzky–Golay smoothing (SG), and its first-derivative (SG-FD) and second-derivative (SG-SD) variants. The application of SPA and CARS to the SVR and BPNN models allowed for the identification of characteristic wavelength variables, resulting in efficient chemometric models for rice adulteration determination. This study can support new insights to enhance the quality assessment of agricultural food products, improving the accuracy and reliability of food detection.

## 2. Materials and Methods

### 2.1. Sampling and Sample Preparation

Two varieties of japonica rice (Wuchang, South japonica, and Song japonica) and four varieties of indica rice (Thai fragrant, Jiangxi silk, and Yunhui) from six rice samples were examined in this study. The rice was classified into high- and low-price categories based on the rice prices in the Chinese market. High-price rice comprised Wuchang rice (Wuchang Golden Harvest Seed Co., Ltd., Wuchang, China) and Thai fragrant rice, while low-price rice included South japonica, Song japonica, Jiangxi silk, and Yunhui rice from Wuxi National Grain Reserve. The study conducted a comprehensive qualitative and quantitative analysis on the issue of adding low-price rice into high-price varieties.

In the japonica category, Wuchang rice served as the reference, with Nanjing and Songjing rice (low-price) added at increasing proportions of 0%, 5%, 10%, 15%, 20%, 25%, 30%, 40%, 50%, and 100% [23]. Similarly, in the indica category, Thai fragrant rice was the reference, with Jiangxi silk and Yunhui rice (low-price) mixed at the same proportions. All mixed samples were ground into a powder and sieved through an 80-mesh standard sieve. A total of 960 samples were prepared, with 210 samples at 0% adulteration and 30 samples per remaining ratio.

### 2.2. Material and Equipment

A self-made quartz cuvette with 2 mm optical path was provided by Yixing Hong Guang Spectrual Instruments Co., Ltd. (Yixing, China). A near-infrared spectrometer (Shanghai FuXiang Instruments Co., Ltd., FX 2000, Shanghai, China) was used to perform the determination.

### 2.3. NIRS Data Acquisition

The samples were collected using an FX 2000 near-infrared spectrometer. The blank collection was used as the measurement background. The samples were weighed and placed in a sample cup to avoid gaps. Morpho (Version 3.2) was used to collect the spectral data of the samples. The instrument process parameters were set. The wavelength-acquisition range was 900~1700 nm, the resolution was 7.8 nm, and the integration time was set to 20 ms. Three parallel spectra were collected for each sample, and each scan was repeated 32 times. The rice samples were placed in the cuvette and scanned three times from three different angles (0°, 120°, and 240°). The average value of the three measurements was then used as the input data for the model to enhance the accuracy of the spectral data.

### 2.4. Sample Set Division

Sample set partitioning was a crucial step in ensuring the performance and accuracy of models used for qualitative and quantitative analysis of NIRS data. It involves dividing the total sample data into a training set and a prediction set. In this study, the spectral sample set was divided using SPXY and KS algorithms in a ratio of 3:1.

### 2.5. Spectral Pre-Treatments

The spectral data were averaged and preprocessed by MATLAB (R2022a); this study employed six spectral pretreatment methods: MSC, SNV, SG, SG-FD, SG-SD, and MMN. SNV was deployed to counteract the influence of particle size variations, surface scattering, and light range discrepancies on diffuse reflectance spectra. MSC targeted the scattering issues resulting from uneven particle distribution and size disparities. SG aimed to address sample background interference, enhance peak separation, and boost sensitivity [9,10]. SG-FD improved the accuracy of corrected spectra, while SG-SD facilitated precise peak positioning in complex peak shapes. The SG filter parameters were set as follows: the polynomial order was 2, and the number of smoothing points used for the local least-squares fit was 5. However, it is crucial to consider that derivative processing can amplify noise, necessitating smoothing of the spectral data before applying it [24].

### 2.6. Model Building

PLSR and SVR were employed for the modeling and analysis of preprocessed full-band spectral data. The models were developed using MATLAB (R2022a). To streamline the process, two feature wavelength screening algorithms, CARS and SPA, were employed to select the most informative and efficient feature variables. Parameter optimization for the SVR model was further enhanced through the utilization of cross-validation (CV), genetic algorithm (GA), and particle swarm optimization (PSO) techniques [25].

A BPNN model was adopted for the qualitative regression analysis. The network architecture consisted of 256 input neurons, followed by three hidden layers with 8, 15, and 9 neurons, respectively, and a single output neuron. The tansig activation function was applied to all hidden layers, while the output layer employed the purelin function for its computations.

### 2.7. Model Evaluation

The correlation coefficient for cross-validation (R^2^c), correlation coefficient for cross-validation (RMSEC), correlation coefficient for prediction (R^2^p), root mean square error for prediction (RMSEP), and ratio of performance to deviation (RPD) were used as evaluation indicators [13,22].

R^2^c and R^2^p represent the correlation degree between the predicted value and the true value; the closer to 1, the better the prediction result. RMSEC and RMSEP are used to evaluate the accuracy of the prediction results of the samples in the calibration set and the prediction set. RMSEC is used to measure the fitting degree of the model to the calibration set. RMSEP is used to measure the generalization ability of the model. In addition, RPD is the inverse of the ratio of RMSEP to STD and is a measure of the predictive power of the model with respect to data variability. A high RPD value means that the model has good predictive power, and it is generally considered that an RPD greater than 2.5 signifies a good predictor, while an RPD of 3 or higher is considered to signify very good predictive performance [10].

## 3. Results and Discussion

### 3.1. NIRS Data for Adulterated Rice

**Figure 1:**
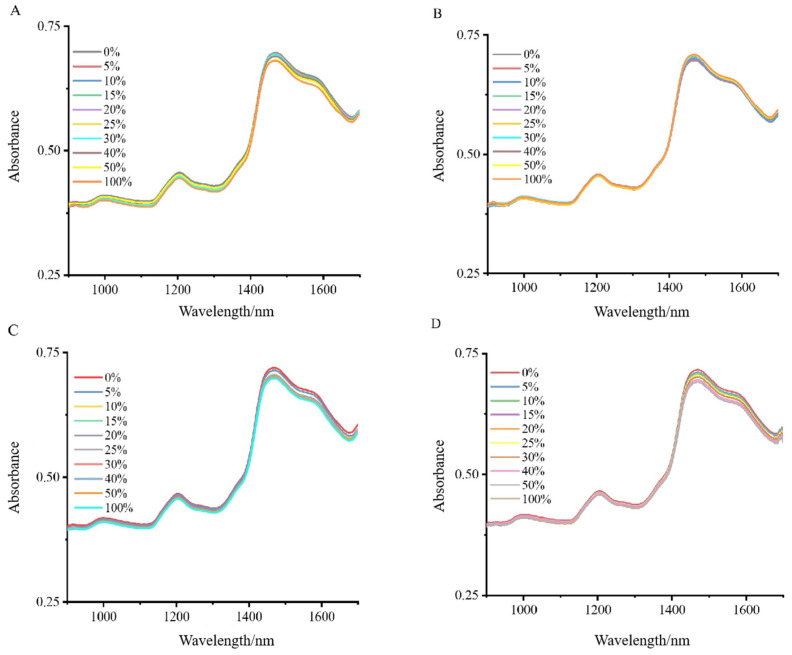
NIRS data analysis of blend proportions: Wuchang and Thai fragrant rice mixed with Nanjing, Songjing, Jiangxi silk, and Yunhui rice: (**A**) NIRS data for Wuchang rice mixed with different proportions of Nanjing; (**B**) NIRS data for Wuchang rice mixed with different proportions of Songjing; (**C**) Thai fragrant rice mixed with different proportions of Jiangxi silk rice; (**D**) Thai fragrant rice mixed with different proportions of Yunhui rice.

The NIRS analysis revealed distinct absorption patterns when Wuchang rice was combined with varying proportions of Nanjing and Songjing rice (Figure 1A,B), and Thai fragrant rice was blended with Yunhui and Jiangxi silk rice (Figure 1C,D). Notably, two prominent peaks were detected: a peak at 1470 nm, corresponding to the first overtone of O-H stretching vibrations, indicative of starch content, and another at 1204 nm, representing secondary C-H bond vibrations, suggesting sugars and starch. Despite the overall high similarity of the spectra, these specific absorption peaks provided key information for distinguishing between different mixing ratios. It is due to these subtle yet important spectral differences that a machine learning approach became necessary for a comprehensive analysis of the NIRS data for the Wuchang–Nanjing–Songjing and Thai fragrant–Jiangxi silk-Yunhui mixtures.

### 3.2. Quantitative Analysis Based on the PLSR Model

#### 3.2.1. Full-Band PLSR Model

For the quantitative regression analysis of high-price rice blended with low-price rice, the collected spectral sample set was divided using the SPXY and KS algorithms. Six preprocessing techniques were applied in combination, and a PLSR model was built. The calibration and prediction sets were analyzed using the LOO-CV algorithm to determine the optimal number of factors. A total of 48 models were created, out of which eight rice models with favorable evaluation results were selected, resulting in 36 models in total.

The analysis of the content of high-price Wuchang rice blended with low-price rice (Nanjing and Songjing) (Table 1) involved the division of calibration and prediction sets using the KS algorithm. After pre-treatment with SNV, the PLSR model was applied to Wuchang rice (high-price) blended with Nanjing rice (low-price), utilizing 17 as the optimal number of factors. KS-SNV-PLSR showed higher R^2^p (0.7945), RPD (1.6734) and smaller RMSEP (0.1506) compared to other models, indicating its superior predictive ability. Other models did not perform as well as the KS-SNV-PLSR model. Similarly, in the analysis of the content of Wuchang rice blended with Songjing rice, the KS-MSC-PLSR model exhibited the best predictive ability.

The KS-MMN-PLSR model showcased exceptional predictive capabilities when analyzing the composition of Thai fragrant rice (high-price) blended with Jiangxi silk rice (low-price), as depicted in Table 1. In the context of detecting Thai fragrant rice (high-price) adulterated with Yunhui rice (low-price), the SPXY-SG-PLS model surpassed other alternatives in its predictive performance. However, the PLSR model fell short in analyzing adulterated low-price rice due to its limited discriminative power in extracting relevant spectral information.

However, it has been found that the original full-band spectral data often contain a lot of redundant information. These unnecessary details can increase the computational complexity. Consequently, feature wavelength screening was essential to eliminate redundant spectral data. In previous studies, a real-time quantitative detection method employing NIRS and chemometrics was developed for rice adulteration, and a PLSR model was optimized through treatments like SNV and MSC [26]. Yu et al. [22] have also reported the performance of feature wavelength screening when NIRS and chemometrics were used for the identification of Tartary buckwheat adulteration.

#### 3.2.2. Non-Full Band PLSR Model

For the analysis of Wuchang rice blended with Nanjing rice, using the SPA, the number of spectral characteristic variables was reduced from an initial 256 to 29 (Table 2). These 29 optimal characteristic variables are at wavelengths of 907.82, 911.15, 921.14, 924.46, 1334.82, 1360.71, 1393.01, 1409.13, 1418.8, 1425.24, 1431.68, 1463.82, 1473.46, 1511.91, 1521.51, 1540.69, 1556.65, 1563.03, 1582.15, 1588.52, 1617.14, 1655.22, 1696.35, 1702.67, 1705.82, 1708.98, 1712.14, 1715.29, and 1718.45 nm. For the analysis of Wuchang rice blended with Nanjing rice, using the CARS, the number of spectral characteristic variables was reduced from an initial 256 to 37. These 37 optimal characteristic variables are at wavelength of 917.81, 951.05, 961.01, 967.64, 997.47, 1027.24, 1043.76, 1060.26, 1073.44, 1103.07, 1126.08, 1132.64, 1135.92, 1168.71, 1198.15, 1204.69, 1224.28, 1234.07, 1237.33, 1263.39, 1273.15, 1302.39, 1315.37, 1338.06, 1341.3, 1360.71, 1376.87, 1386.56, 1389.78, 1393.01, 1418.8, 1450.98, 1473.46, 1483.08, 1582.15, 1626.67, and 1636.19 nm. However, the PLSR model performed better when applied to the full band rather than the non-full band using SPA and CARS algorithms, indicating that valuable information was lost during wavelength screening, which negatively impacted the model’s overall effectiveness. Consequently, the KS-SNV-PLSR model emerged as the optimal quantitative approach for analyzing the content of Wuchang rice blended with Nanjing. Similarly, the KS-MSC-CARS-PLSR model was the preferred choice for Wuchang rice blended with Songjing. For Thai fragrant rice blended with Jiangxi silk rice, the KS-MMN-PLSR model, employing the PLSR method, showcased excellent performance. The SPXY-SG-CARS-PLSR model successfully addressed the content analysis of Thai fragrant rice blended with Yunhui rice, benefiting from PLSR, SPA, and CARS algorithms for model simplification and elimination of redundant information.

**Figure 2:**
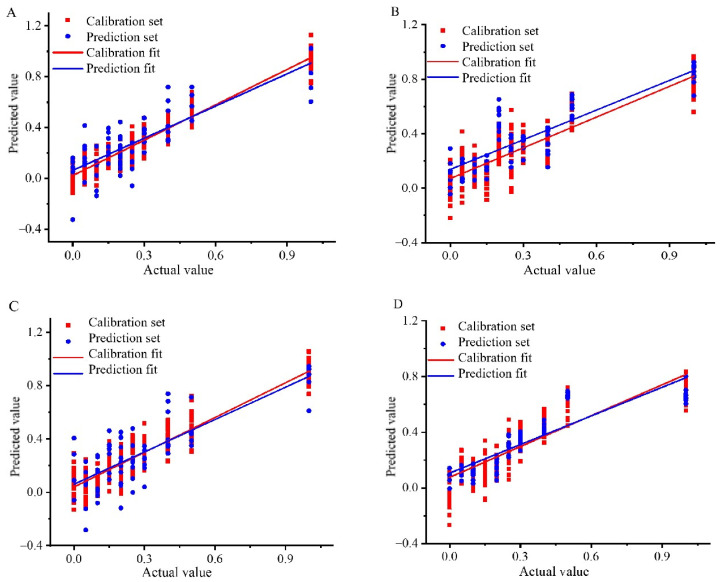
Correlation coefficient diagram of Wuchang rice blended with Nanjing rice: (**A**) Content analysis based on KS-SNV-PLSR model, Wuchang rice blended with Songjing rice; (**B**) Content analysis based on KS-MSC-CARS-PLSR model, Thai fragrant rice blended with Jiangxi silk rice; (**C**) Content analysis based on KS-MMN-PLSR model, Thai fragrant rice blended with Yunhui rice; (**D**) Content analysis based on SPXY-SG-CARS-PLSR model.

Based on these findings, the KS-SNV-PLSR and KS-MSC-CARS-PLSR models were identified as the top quantitative tools for analyzing the content of Wuchang rice blended with Nanjing and Songjing, effectively tackling the challenge of low-price rice in high-price blends. The KS-MMN-PLSR and SPXY-SG-CARS-PLSR models were recognized as the best for Thai fragrant rice adulteration cases involving Jiangxi silk and Yunhui rice. Figure 2 highlights the noticeable difference between the calibration and prediction sets for the best model, indicating that the PLSR model’s inability to capture the dataset’s general pattern might be attributed to data complexity and an algorithm mismatch [27,28,29].

### 3.3. Quantitative Analysis Based on the SVR Model

#### 3.3.1. Full-Band SVR Model

To conduct a quantitative regression analysis on the blending of low-price and high-price rice, the spectral dataset was divided using SPXY and KS algorithms. Six preprocessing techniques, comprising MMN, SG, SNV, SG-FD, SG-SD, and MSC, were employed. The SVR model was employed for modeling and analysis, with the optimal parameters (C, g) selected through CV, GA, and PSO in conjunction with the radial basis function (RBF) kernel. A total of 144 models were constructed, and 14 rice-specific models with superior evaluation metrics were chosen, resulting in a final set of 56 models.

For the content assessment of Wuchang rice blended with Nanjing rice (Table 3), the SPXY algorithm was used to partition the calibration and prediction sets. Following MSC preprocessing and parameter optimization via CV, the SVR model achieved an excellent performance with an R^2^p of 0.9467,an RMSEP of 0.0005 and an RPD of 4.3287. The optimal parameters were found to be (22.6274, 1024). Among the remaining 13 models, the SPXY-MSC-CV-SVR model exhibited the highest accuracy.

In the study of Wuchang rice blended with Songjing rice (Table 3), the KS algorithm was employed for dataset division. After MSC preprocessing and CV parameter tuning, the SVR model demonstrated a predictive power of R^2^p (0.8945), RMSEP (0.0092) and RPD (2.9187), with the best parameter set at (45.2548, 1024). Compared to the other 13 models, the SPXY-MSC-CV-SVR model stood out in terms of predictive accuracy.

In the analysis of Thai fragrant rice blended with Jiangxi silk rice (Table 3), the KS algorithm was used to divide the calibration and prediction sets. Applying SNV preprocessing and CV parameter optimization, the SVR model achieved an R^2^p of 0.8077, an RMSEP of 0.012 and an RPD of 2.2805, with the best combination of parameters determined as (11.3137, 32). When compared to the other 13 models, the KS-SNV-CV-SVR model demonstrated high accuracy and good predictive power. From the data presented in Table 3, it is evident that, in the analysis of Thai fragrant rice blended with Yunhui rice, the calibration and prediction sets were divided using the KS algorithm. The SVR model achieved an R^2^p of 0.8647, an RMSEP of 0.0321 and an RPD of 1.8133 after determining the best combination of parameters as (16, 1024) with MSC preprocessing and CV parameter optimization. The KS-MSC-CV-SVR model outperformed the other 13 models, indicating superior predictive capabilities. Previous research has demonstrated that MSC effectively mitigates spectral discrepancies in powdered sample measurements, enhancing the signal-to-noise ratio [30]. A comparison revealed that the SVR-based discrimination model surpassed PLSR in overall performance, substantiating SVR’s efficacy in addressing challenges such as small sample size, nonlinearity, multidimensionality, and local minima. SVR’s superior performance in these scenarios ensures greater reliability, particularly with limited data [7].

#### 3.3.2. Non-Full Band SVR Model

For the analysis of Wuchang rice blended with Nanjing rice, using the SPA, the number of spectral characteristic variables was reduced from an initial 256 to 16 (Table 4). These 16 optimal characteristic variables are at wavelengths of 897.83, 907.82, 921.14, 924.46, 1139.21, 1334.82, 1360.71, 1393.01, 1473.46, 1479.87, 1540.69, 1569.41, 1588.52, 1598.07, 1652.05, and 1712.14 nm. Using the CARS, the number of spectral characteristic variables was reduced from an initial 256 to 41. These 37 optimal characteristic variables are at wavelength of 897.83, 907.82, 924.46, 931.11, 937.76, 944.41, 951.05, 967.64, 987.54, 1020.63, 1023.94, 1027.24, 1033.85, 1060.26, 1080.03, 1089.91, 1103.07, 1122.79, 1126.08, 1129.36, 1135.92, 1139.21, 1149.05, 1168.71, 1198.15, 1240.59, 1289.4, 1308.88, 1321.86, 1331.58, 1338.06, 1360.71, 1363.94, 1396.24, 1473.46, 1476.66, 1483.08, 1489.49, 1543.88, 1582.15, and 1598.07 nm.

The SVR model surpassed the SPA and CARS-based models in the content analysis of the Wuchang and Nanjing rice blend, with lower RMSEC and RMSEP values compared to the full band SVR model. The most effective quantitative models for this blend were SPXY-MSC-CV-SVR and KS-MSC-CV-SVR. In the content analysis of Thai fragrant rice mixed with Jiangxi silk rice, the full band SVR model outperformed the non-full band using SPA, but the non-full band exhibited better overall performance, accounting for less than 14.1% of total variance when employing CARS. Consequently, the optimal model for this combination was KS-SNV-CV-CARS-SVR. In a previous study, SPA was successfully employed to identify hawthorn leaf origins, highlighting the advantage of feature wavelength selection in extracting essential information and improving model efficiency and accuracy, supporting the applicability of this approach in similar spectral analyses [31].

The SVR model outperformed non-full band methods, such as SPA and CARS, in the analysis of Thai fragrant rice blended with Yunhui rice. Consequently, the KS-MSC-CV-SVR model emerged as the most effective quantitative approach. Previous research also found SVR’s full-spectrum model to be more effective than its non-full-spectrum counterpart in assessing adulterated rice oil. The non-full-spectrum’s inferior performance might be attributed to the potential loss of crucial information during feature wavelength selection or inadequate extraction of relevant data during the qualitative analysis.

**Figure 3:**
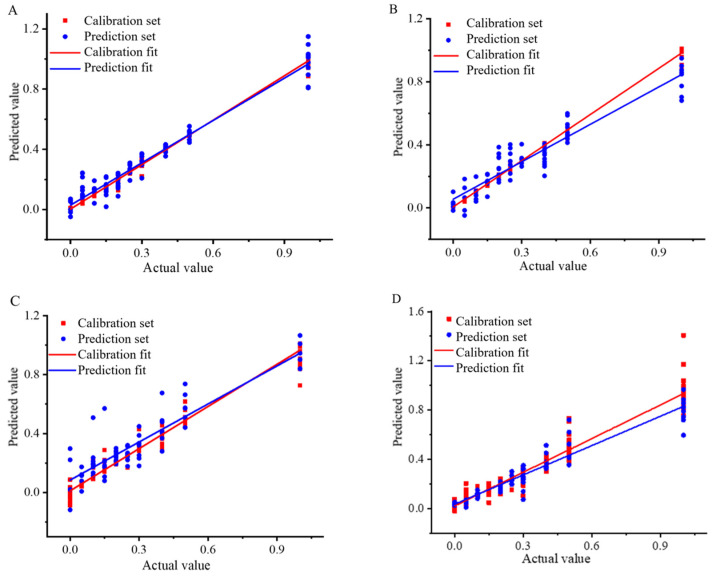
Correlation coefficient diagram of Wuchang rice blended with Nanjing rice: (**A**) Content analysis based on SPXY-MSC-CV-SVR model, Wuchang rice blended with Songjing rice; (**B**) Content analysis based on KS-MSC-CV-SVR model, Thai fragrant rice blended with Jiangxi silk rice; (**C**) Content analysis based on KS-SNV-CV-CARS-SVR model, Thai fragrant rice blended with Yunhui rice; (**D**) Content analysis based on KS-MSC-CV-SVR model.

From the above study, it was evident that the best quantitative models for analysis of Wuchang rice blended with Nanjing rice and Songjing rice were SPXY-MSC-CV-SVR and KS-MSC-CV-SVR. These models utilized the SVR model and demonstrated favorable evaluation indexes such as R^2^p, RMSEP and RPD. For the analysis of Thai fragrant rice blended with Jiangxi silk rice and Yunhui rice, the optimal models were KS-SNV-CV-CARS-SVR and KS-MSC-CV-SVR, with excellent agreement between the calibration and prediction sets (Figure 3).

### 3.4. Quantitative Analysis Based on the BPNN Model

A comprehensive BPNN model was employed to analyze doped low-price rice, comparing various BPNN-based models alongside diverse sample division and preprocessing techniques. The spectral dataset of Wuchang rice blended with Nanjing rice was classified using SPXY and KS algorithms, followed by preprocessing. The BPNN model was then applied to calibration and prediction subsets, resulting in 48 models. Six high-performing models were selected, totaling 24 models.

It was revealed that the SPXY-SG-BPNN model exhibited superior predictive capabilities for Wuchang rice blended with both Nanjing rice and Songjing rice, achieving an R^2^p of 0.9055, an RMSEP of 0.1235 and an RPD of 2.3462. When Wuchang rice was mixed with Nanjing rice, the SPXY algorithm was used for set division, while for Wuchang–Japonica mixtures, the same algorithm was employed. The MMN-preprocessed BPNN model surpassed other models, showcasing the highest predictive power.

According to the data in Table 5, the analysis of the content of Thai fragrant rice blended with low-price Jiangxi silk rice and Yunhui rice showed promising results. When Thai fragrant rice was blended with Jiangxi silk rice, the KS algorithm was employed for division, and the BPNN model, pre-treated with SNV, achieved an R^2^p of 0.8775, an RMSEP of 0.1225 and an RPD of 2.0570, with superior predictive ability. Moreover, for Thai fragrant rice blended with Yunhui rice, the SPXY algorithm was used for division, and the BPNN model, pre-treated with SG, yielded an R^2^p of 0.9496, an RMSEP of 0.0963 and an RPD of 3.0049, surpassing other models and demonstrating the best predictive capability. Our findings were similar to those of previous research that found BPNN performed well in quantitative analysis of low-price rice due to its ability to extract feature wavelengths for analysis, resulting in accurate predictions [32].

### 3.5. Comparative Analysis of Quantitative Models

**Figure 4:**
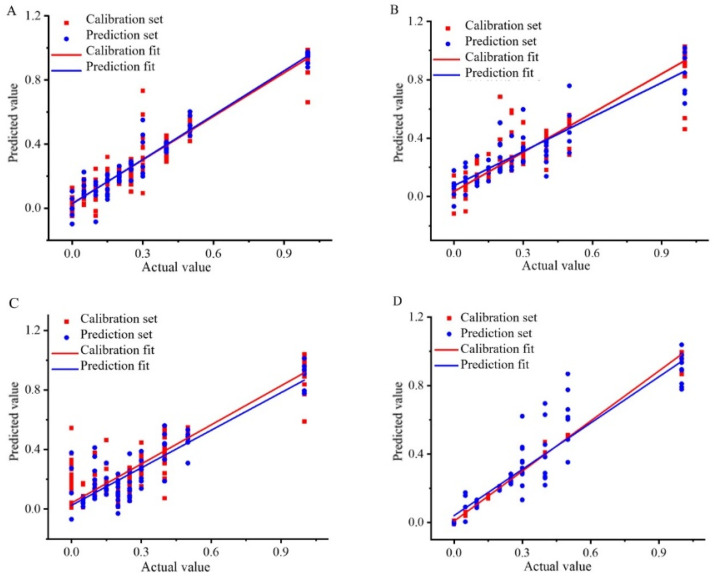
Correlation coefficient diagram of Wuchang rice blended with Nanjing rice: (**A**) Content analysis based on SPXY-SG-BPNN model, Wuchang rice blended with Songjing rice; (**B**) Content analysis based on SPXY-MMN-BPNN model, Thai fragrant rice blended with Jiangxi silk rice; (**C**) Content analysis based on KS-SNV-BPNN model, Thai fragrant rice blended with Yunhui rice; (**D**) Content analysis based on SPXY-SG-BPNN model.

In the content analysis of Wuchang rice blended with Nanjing rice and Songjing rice, the KS-SNV-PLSR, SPXY-MSC-CV-SVR, and SPXY-SG-BPNN models emerged as the best for PLSR, SVR, and BPNN, respectively. The SPXY-SG-BPNN model demonstrated superior prediction capabilities, as illustrated in Figure 4A, which showcases the correlation coefficients for Wuchang–Nanjing content analysis. Similarly, for Wuchang–Songjing mixtures, the KS-MSC-CARS-PLSR, KS-MSC-CV-SVR, and SPXY-MMN-BPNN models stood out, with the SPXY-MMN-BPNN model offering the best prediction results (Figure 4B, which displays the correlation coefficients after SPXY-MMN-BPNN analysis).

After conducting a content analysis of Thai fragrant rice blended with Jiangxi silk rice and Yunhui rice, the most effective models were identified as KS-MMN-PLSR for PLS, KS-SNV-CV-CARS-SVR for SVR, and KS-SNV-BPNN for BPNN. The KS-SNV-BPNN model demonstrated superior accuracy in predictions (Figure 4C, which displays the correlation coefficients for the rice content analysis of the mixed Thai fragrant and Jiangxi silk rice). Similarly, when Thai fragrant rice was blended with Yunhui rice, the best models were SPXY-SG-CARS-PLSR for PLSR, KS-MSC-CV-SVR for SVR, and SPXY-SG-BPNN for BPNN. The SPXY-SG-BPNN model provided the most reliable predictions, and Figure 4D presents the correlation coefficients for the rice content analysis of high-price Thai fragrant rice contaminated with Yunhui rice.

The prediction performance of PLSR was better than SVR for the mixture of Wuchang, Nanjing and Songjing rices with different ratios. However, for the mixtures of Thai fragrant rice with different ratios of Yunhui rice and Jiangxi silk rice, SVR provided more accurate results because of the differences in chemical components in the samples. The mixture of Wuchang with Nanjing and Songjing rices mainly depends on the change of starch content, which has a clear absorption peak at 1470 nm [33]. PLSR is good at dealing with linear relationships and can effectively capture the changes at this critical wavelength, thus showing good predictive ability for these kinds of mixture [34,35]. In contrast, the mixture of Thai fragrance rice with Yunhui rice and Jiangxi silk rice involved not only changes in starch content but also changes in the proportion of other components such as sugars, which were also reflected at 1204 nm [36]. SVR performs well in dealing with nonlinear problems and can better adapt to these complex spectral characteristics, and it performs better in analyzing Thai fragrant rice with added low-price rice [37,38].

As above, the NIRS combined with chemometrics demonstrated its efficacy in detecting low-price rice blended with high-price rice. The RPD value of low-price rice blended with high-price rice for our model indicates a strong predictive capability, suggesting that the model performs well in predicting the adulterated rice content. This high RPD value also demonstrates the robustness of our model across different datasets, enhancing confidence in its generalizability [24]. The SPXY algorithm generally showcased strong performance across most scenarios, whereas the KS algorithm exhibited superior results specifically for Thai fragrant rice contaminated with Jiangxi silk rice. Consequently, the SPXY algorithm is more appropriate for content analysis of low-price rice mixed with high-price rice. Among various pre-treatments, MSC, SNV, and SG stood out for enhancing the signal-to-noise ratio and minimizing spectral variations in powder samples. The SVR and BPNN models outperformed PLSR in assessing the content of blended low-price rice. Notably, the BPNN model, known for its inherent feature extraction capabilities, proved particularly adept at predicting the adulterated rice content [39].

## 4. Conclusions

This study utilized NIRS chemometrics to quantify the adulteration of low-price rice with high-price rice. Predictive models were constructed using PLSR, SVR, and BPNN algorithms. Six preprocessing methods, along with CARS and SPA feature selection techniques, and three parameter-optimization algorithms were applied to analyze 10 samples of adulterated high-price rice. The resulting models, namely SPXY-SG-BPNN, SPXY-MMN-BPNN, KS-SNV-BPNN, and SPXY-SG-BPNN, demonstrated high accuracy in identifying Wuchang rice blended with japonica rice, Thai fragrant rice with indica, and Thai fragrant rice with Yunhui rice. The BPNN models achieved correlation coefficients exceeding 87%, indicating a high level of accuracy for determining the degree of adulteration in high-price rice. Consequently, NIRS has emerged as a promising technique for the rapid, non-destructive detection of low-price rice in high-price rice mixtures. Future research should focus on enhancing the universality and practicality of these models, and developing new models for real sample applications. Additionally, incorporating a diverse range of algorithms, including deep learning, could further improve model analysis and comparison.

## Figures and Tables

**Table 1:** PLSR model for rice content analysis of high-price rice (Wuchang rice and Thai fragrant rice) blended with low-price rice (Nanjing rice, Songjing rice, Jiangxi silk seedling rice, and Yunhui rice) using different sample set partitioning methods.

Sample Set Division Method	Preprocessing	Optimal Number of Factors	Correction Set		Prediction Set		
			R^2^c	RMSEC	R^2^p	RMSEP	RPD
Wuchang rice blended with Nanjing rice							
SPXY	MSC	14	0.9313	0.0917	0.7442	0.1829	1.6526
	SNV	16	0.9538	0.0771	0.7807	0.1749	1.7265
	SG	13	0.8667	0.1196	0.7542	0.1689	1.7894
	SG-FD	15	0.9482	0.0811	0.7902	0.1757	1.7238
KS	MMN	15	0.9352	0.0961	0.7502	0.1529	1.5484
	SNV	17	0.9600	0.0776	0.7945	0.1506	1.6734
	SG-FD	15	0.9502	0.0857	0.7588	0.1646	1.4670
	SG-SD	10	0.9145	0.1080	0.7853	0.1413	1.7156
Wuchang rice blended with Songjing rice							
SPXY	MMN	10	0.8371	0.1308	0.4477	0.1844	1.5770
	SG	11	0.7916	0.1421	0.4778	0.1797	1.6180
KS	MMN	8	0.7883	0.1446	0.6763	0.1673	1.6616
	MSC	9	0.8068	0.1403	0.7057	0.1655	1.6827
	SNV	9	0.8074	0.1402	0.7022	0.1654	1.6831
	SG	11	0.7783	0.1467	0.6911	0.1631	1.7119
	SG-FD	12	0.9011	0.1098	0.6969	0.1727	1.6087
	SG-SD	9	0.8858	0.1161	0.6523	0.1816	1.5348
Thai fragrant rice blended with Jiangxi silk seedling rice							
SPXY	MMN	14	0.8852	0.1227	0.7010	0.1573	1.4387
	MSC	15	0.9068	0.1130	0.7380	0.1515	1.4963
	SNV	15	0.9075	0.1127	0.7398	0.1515	1.4957
	SG	13	0.8403	0.1387	0.7194	0.1535	1.4757
KS	MMN	17	0.9213	0.1036	0.7698	0.1617	1.5370
	MSC	15	0.9084	0.1103	0.7495	0.1599	1.5546
	SNV	15	0.9090	0.1100	0.7501	0.1600	1.5540
	SG-SD	11	0.8985	0.1149	0.6724	0.1827	1.4236
Thai fragrant rice blended with Yunhui rice							
SPXY	MMN	7	0.7184	0.1585	0.5779	0.1731	1.5857
	MSC	6	0.7012	0.1611	0.5764	0.1694	1.6202
	SNV	6	0.7018	0.1610	0.5845	0.1693	1.6210
	SG	8	0.8040	0.1419	0.7471	0.1409	1.9502
	SG-FD	5	0.7501	0.1531	0.7254	0.1471	1.8648
	SG-SD	7	0.7628	0.1507	0.6778	0.1546	1.7735
KS	SG	7	0.8047	0.1381	0.5723	0.1612	1.7907
	SG-SD	5	0.7663	0.1462	0.5642	0.1670	1.7310

**Table 2:** Optimization results of PLSR model for analyzing the content of high-price rice (Wuchang rice and Thai fragrant rice) blended with low-price rice (Nanjing rice, Songjing rice, Jiangxi silk seedling rice, and Yunhui rice) using SPA and CARS algorithms.

Types	Models	Number of Wavelengths	Optimal Number of Factors	Correction Set		Prediction Set		
				R^2^c	RMSEC	R^2^p	RMSEP	RPD
Wuchang rice blended with Nanjing rice and Songjing rice								
Nanjing	KS-SNV-PLSR	256	17	0.9600	0.0776	0.7945	0.1506	1.6734
	KS-SNV-SPA-PLSR	29	20	0.8486	0.1346	0.6810	0.1562	1.4482
	KS-SNV-CARS-PLSR	37	10	0.8987	0.1156	0.7847	0.1471	1.6018
Songjing	KS-MSC-PLSR	256	9	0.8068	0.1403	0.7057	0.1655	1.6827
	KS-MSC-SPA-PLSR	10	9	0.7302	0.1556	0.5203	0.1809	1.4531
	KS-MSC-CARS-PLSR	26	8	0.8196	0.1371	0.7537	0.1576	1.8531
Thai fragrant rice blended with Jiangxi silk rice and Yunhui rice								
Jiangxi silk	KS-MMN-PLSR	256	17	0.9213	0.1036	0.7698	0.1617	1.5370
	KS-MMN-SPA-PLSR	27	17	0.7440	0.1588	0.6533	0.1858	1.3447
	KS-MMN-CARS-PLSR	44	11	0.8870	0.1198	0.7591	0.1694	1.4674
Yunhui	SPXY-SG-PLSR	256	8	0.8040	0.1419	0.7471	0.1409	1.9502
	SPXY-SG-SPA-PLSR	5	4	0.7692	0.1495	0.7524	0.1347	2.0242
	SPXY-SG-CARS-PLSR	40	7	0.8051	0.1416	0.7769	0.1352	2.3242

**Table 3:** SVR models for rice content analysis of high-price rice (Wuchang rice and Thai fragrant rice) blended with low-price rice (Nanjing rice, Songjing rice, Jiangxi silk seedling rice, and Yunhui rice) using different sample set partitioning methods.

Sample Set Division	Parameter Optimization	Preprocessing	Optimal Parameters		Correction Set		Prediction Set		
			C	g	R^2^c	RMSEC	R^2^p	RMSEP	RPD
Wuchang rice blended with Nanjing rice									
SPXY	CV	MMN	45.2548	45.2548	0.9959	0.0003	0.9326	0.0062	3.8488
		MSC	22.6274	1024	0.9961	0.0003	0.9467	0.005	4.3287
		SNV	22.6274	11.3137	0.9958	0.0003	0.9456	0.0051	4.2876
		SG	8	90.5097	0.8968	0.0074	0.9259	0.0068	3.6651
	GA	MSC	23.9151	380.6086	0.9694	0.0023	0.9002	0.0092	3.1384
	PSO	MSC	37.7343	381.0129	0.9797	0.0016	0.9035	0.0089	3.2009
		SNV	14.7347	4.4589	0.9583	0.0031	0.8980	0.0094	3.1068
KS	CV	MMN	22.6274	64	0.9929	0.0006	0.9212	0.0047	3.4779
		MSC	22.6274	1024	0.9961	0.0003	0.9190	0.0007	2.9990
		SNV	22.6274	11.3137	0.9957	0.0004	0.9184	0.0048	3.4640
		SG	1024	32	0.9739	0.0022	0.8972	0.006	3.0936
	GA	MSC	50.4882	482.5373	0.9923	0.0007	0.9057	0.0064	3.0314
		SNV	48.4435	5.4951	0.9918	0.0007	0.9056	0.0057	3.1934
	PSO	SNV	7.4899	5.4347	0.9413	0.0051	0.8965	0.0066	2.9471
Wuchang rice blended with Songjing rice									
SPXY	CV	MMN	11.3137	128	0.9861	0.0011	0.8526	0.0127	2.6017
		MSC	11.3137	1024	0.9639	0.003	0.8888	0.0125	2.6413
		SNV	4	32	0.9763	0.002	0.8814	0.0101	2.8988
		SG	1024	16	0.8510	0.0121	0.7837	0.0210	2.0398
		SG-FD	1024	1024	0.9286	0.006	0.7428	0.0224	1.9317
	GA	MMN	33.7168	16.2077	0.8715	0.0105	0.7854	0.0190	2.1051
		MSC	19.9383	352.4494	0.8931	0.0089	0.8227	0.0180	2.2470
		SNV	3.8597	4.4051	0.7260	0.0249	0.6764	0.0314	1.6866
	PSO	SNV	7.0270	4.6253	0.8103	0.0166	0.7555	0.0228	1.9514
KS	CV	MMN	16	90.5097	0.9768	0.002	0.8716	0.0103	2.7419
		MSC	45.2548	1024	0.9965	0.0003	0.8945	0.0092	2.9187
		SNV	16	22.6274	0.9955	0.0004	0.8845	0.0091	2.9287
		SG	724.077	11.3137	0.7712	0.0187	0.7201	0.0235	1.8407
	PSO	SNV	33.9274	2.6117	0.8530	0.0119	0.7362	0.0209	1.9402
Thai fragrant rice blended with Jiangxi silk rice									
SPXY	CV	MSC	90.5097	1024	0.9950	0.0004	0.8031	0.0103	2.2348
		SNV	32	22.6274	0.9950	0.0004	0.8014	0.0103	2.2266
		SG	1024	4	0.6465	0.0343	0.7520	0.0147	1.8812
	GA	MMN	29.6159	23.387	0.7618	0.029	0.7222	0.0157	1.8034
		MSC	82.1792	175.849	0.8448	0.0192	0.7331	0.0141	1.9081
		SNV	81.6226	1.9465	0.8287	0.0218	0.7294	0.0146	1.8763
		SG	90.8217	18.1971	0.5709	0.0414	0.7689	0.0160	1.8158
	PSO	MMN	27.1091	24.6807	0.7578	0.0294	0.7215	0.0158	1.7992
		MSC	85.0057	171.7987	0.8454	0.0191	0.7325	0.0141	1.9070
		SNV	57.9140	2.1146	0.7886	0.0270	0.7220	0.0157	1.8091
		SG	83.4726	16.1597	0.5457	0.0441	0.7540	0.0174	1.7467
KS	CV	MMN	32	128	0.9934	0.0006	0.7593	0.0153	2.0362
		MSC	45.2548	1024	0.981	0.0016	0.7956	0.0224	2.0731
		SNV	11.3137	32	0.9847	0.0013	0.8077	0.0121	2.2805
Thai fragrant rice blended with Yunhui rice									
SPXY	CV	MMN	2	362.0387	0.9506	0.0041	0.8293	0.0138	2.4152
		MSC	16	1024	0.9433	0.0047	0.7829	0.0192	2.1243
		SNV	2.8284	90.5097	0.9808	0.0015	0.8547	0.0114	2.6221
		SG	1024	8	0.8641	0.0114	0.8068	0.0162	2.2092
	GA	MMN	45.2050	11.5872	0.8324	0.0174	0.7166	0.0255	1.7996
		MSC	29.9354	117.2724	0.7865	0.0225	0.7189	0.0275	1.7279
		SG	65.5509	1.5603	0.7757	0.0181	0.7841	0.0182	2.0823
		SG-FD	90.6014	259.2163	0.7481	0.0257	0.7136	0.0284	1.6952
	PSO	SG	30.737	2.6073	0.7751	0.0182	0.7824	0.0184	2.0735
		SG-FD	84.7656	279.656	0.7507	0.0255	0.7143	0.0283	1.6781
KS	CV	MMN	5.6569	512	0.9968	0.0003	0.8531	0.0125	2.5846
		MSC	16	1024	0.9338	0.0058	0.8647	0.0321	1.8133
		SNV	2.8284	90.5097	0.985	0.0012	0.8599	0.0121	2.6302
		SG	1024	16	0.9265	0.006	0.7626	0.0205	2.0277

**Table 4:** SVR models for analyzing the content of high-price rice (Wuchang rice and Thai fragrant rice) blended with low-price rice (Nanjing rice, Songjing rice, Jiangxi silk seedling rice, and Yunhui rice) using SPA and CARS algorithms.

Types	Models	Number of Wavelengths	Optimal Parameters		Correction Set		Prediction Set		
			C	g	R^2^c	RMSEC	R^2^p	RMSEP	RPD
Wuchang rice blended with Nanjing rice and Songjing rice									
Nanjing	SPXY-MSC-CV-SVR	256	22.6274	1024	0.9961	0.0003	0.9467	0.005	4.3287
	SPXY-MSC-CV-SPA-SVR	16	1024	1024	0.9391	0.0046	0.8578	0.0133	1.2562
	SPXY-MSC-CV-CARS-SVR	41	1024	1024	0.9520	0.0035	0.8940	0.0116	1.4355
Songjing	KS-MSC-CV-SVR	256	45.2548	1024	0.9965	0.0003	0.8945	0.0092	2.6413
	KS-MSC-CV-SPA-SVR	10	8	362.0387	0.8943	0.0083	0.7232	0.0217	1.8920
	KS-MSC-CV-CARS-SVR	26	90.5097	1024	0.8077	0.0156	0.7287	0.0520	1.8848
Thai fragrant rice blended with Jiangxi silk rice and Yunhui rice									
Jiangxi Silk	KS-SNV-CV-SVR	256	11.3137	32	0.9847	0.0013	0.8077	0.0121	2.2805
	KS-SNV-CV-SPA-SVR	45	16	90.5097	0.9525	0.0040	0.7695	0.0156	2.0520
	KS-SNV-CV-CARS-SVR	30	5.6569	1024	0.9834	0.0014	0.8220	0.0131	2.3574
Yunhui	KS-MSC-CV-SVR	256	16	1024	0.9338	0.0058	0.8647	0.0321	1.8133
	KS-MSC-CV-SPA-SVR	5	1024	1024	0.7038	0.0255	0.7118	0.0544	1.6712
	KS-MSC-CV-CARS-SVR	34	64	1024	0.7624	0.0221	0.6811	0.0464	1.5415

**Table 5:** Results of BPNN model for rice content analysis of high-price rice (Wuchang rice and Thai fragrant rice) blended with low-price rice (Nanjing rice, Songjing rice, Jiangxi silk seedling rice, and Yunhui rice) using different sample set partitioning methods.

Sample Set Division Method	Preprocessing	Correction Set		Prediction Set		
		R^2^c	RMSEC	R^2^p	RMSEP	RPD
Wuchang rice blended with Nanjing rice						
SPXY	SNV	0.9887	0.0404	0.9091	0.1260	2.3997
	SG	0.9666	0.0692	0.9729	0.0703	4.2969
	SG-FD	0.9870	0.0431	0.9514	0.0931	3.2478
KS	MMN	0.9681	0.0723	0.9477	0.0764	3.1288
	SNV	0.9947	0.0299	0.9688	0.0599	4.0286
	SG	0.9828	0.0532	0.9437	0.0793	3.0122
Wuchang rice blended with Songjing rice						
SPXY	MMN	0.9513	0.0842	0.9055	0.1235	2.3462
	SG	0.8649	0.1462	0.8527	0.1688	1.7163
	SG-FD	0.9329	0.1105	0.8567	0.1526	1.9003
	SG-SD	0.9330	0.0989	0.8527	0.1548	1.8716
KS	SNV	0.9597	0.0783	0.8519	0.1491	1.8634
	SG-FD	0.9504	0.0872	0.8241	0.1657	1.6924
Thai fragrant rice blended with Jiangxi silk seedling rice						
SPXY	MSC	0.9235	0.1201	0.8482	0.1305	1.7540
	SNV	0.9637	0.0787	0.8602	0.1239	1.9379
KS	MMN	0.9459	0.0943	0.8394	0.1398	1.7831
	SNV	0.9269	0.1074	0.8775	0.1225	2.0570
	SG	0.9378	0.1042	0.8449	0.1352	1.8561
	SG-SD	0.9015	0.1254	0.8715	0.1308	1.5823
Thai fragrant rice blended with Yunhui rice						
SPXY	MMN	0.8881	0.1484	0.8433	0.1833	1.7068
	SNV	0.9613	0.0796	0.8767	0.1354	2.0491
	SG	0.9731	0.0649	0.9496	0.0963	3.0049
	SG-FD	0.9463	0.0897	0.8738	0.1359	2.0281
KS	SNV	0.9397	0.0930	0.8749	0.1442	2.0108
	SG	0.9814	0.0521	0.9338	0.1055	2.7382

## Data Availability

The original contributions presented in the study are included in the article, further inquiries can be directed to the corresponding author.
